# An Analytical Framework of Tonal and Rhythmic Hierarchy in Natural Music Using the Multivariate Temporal Response Function

**DOI:** 10.3389/fnins.2021.665767

**Published:** 2021-07-16

**Authors:** Jasmine Leahy, Seung-Goo Kim, Jie Wan, Tobias Overath

**Affiliations:** ^1^Department of Psychology and Neuroscience, Duke University, Durham, NC, United States; ^2^Department of Cognitive Sciences, University of California, Irvine, Irvine, CA, United States; ^3^Duke Institute for Brain Sciences, Duke University, Durham, NC, United States; ^4^Center for Cognitive Neuroscience, Duke University, Durham, NC, United States

**Keywords:** linearized encoding analysis, electroencephalography, tonal hierarchy, rhythmic hierarchy, naturalistic paradigm

## Abstract

Even without formal training, humans experience a wide range of emotions in response to changes in musical features, such as tonality and rhythm, during music listening. While many studies have investigated how isolated elements of tonal and rhythmic properties are processed in the human brain, it remains unclear whether these findings with such controlled stimuli are generalizable to complex stimuli in the real world. In the current study, we present an analytical framework of a linearized encoding analysis based on a set of music information retrieval features to investigate the rapid cortical encoding of tonal and rhythmic hierarchies in natural music. We applied this framework to a public domain EEG dataset (OpenMIIR) to deconvolve overlapping EEG responses to various musical features in continuous music. In particular, the proposed framework investigated the EEG encoding of the following features: *tonal stability*, *key clarity*, *beat*, and *meter*. This analysis revealed a differential spatiotemporal neural encoding of *beat* and *meter*, but not of *tonal stability* and *key clarity*. The results demonstrate that this framework can uncover associations of ongoing brain activity with relevant musical features, which could be further extended to other relevant measures such as time-resolved emotional responses in future studies.

## Introduction

Music is a universal auditory experience known to evoke intense feelings. Even without musical training, humans not only connect to it on an emotional level but can also generate expectations as they listen to it (Koelsch et al., 2000). We gather clues from what we are listening to in real-time combined with internalized musical patterns, or schema, from our respective cultural settings to guess what will happen next, which ultimately results in a change in our emotions. Schemata consist of musical features, such as tonality (i.e., pitches and their relationship to one another) and rhythm. However, tonality has often been studied using heavily contrived chord progressions instead of more natural, original music in order to impose rigorous controls on the experiment (Fishman et al., 2001; Loui and Wessel, 2007; Koelsch and Jentschke, 2010). Likewise, beat perception studies have favored simplistic, isolated rhythms over complex patterns found in everyday music (Snyder and Large, 2005; Fujioka et al., 2009). Therefore, designs that take advantage of the multiple, complex features of natural music stimuli are needed to confirm the results of these experiments.

In order to devise a framework that can account for these complexities, we first considered how different musical features build up over the course of a piece of music. In everyday music, tonality and rhythm are constructed hierarchically, meaning some pitches in certain positions (e.g., in a bar) have more importance than others (Krumhansl and Shepard, 1979). One way that listeners assess this importance is *via* the temporal positions of pitches. Tones that occur at rhythmically critical moments in a piece allow us to more easily anticipate what we should hear next and when (Krumhansl, 1990; Krumhansl and Cuddy, 2010). This type of beat perception is considered hierarchical in the sense that it involves multiple layers of perception which interact with one another, namely beat and meter. Beat refers to the onset of every beat in a given measure, whereas meter refers to the importance of the beats relative to a given time signature (e.g., 4/4). Music listening has repeatedly been linked with activation of the motor cortex, in particular relating to anticipation of the beat (Zatorre et al., 2007; Chen et al., 2008; Gordon et al., 2018). The clarity of the beat matters during music perception as well; during moments of high beat saliency, functional connectivity increases from the basal ganglia and thalamus to the auditory and sensorimotor cortices and cerebellum, while low beat saliency correlates with increased connectivity between the auditory and motor cortices, indicating that we participate in an active search to find the beat when it becomes less predictable (Toiviainen et al., 2020). EEG studies, in particular, have shed light on how humans entrain beat and meter on both a micro-scale (e.g., milliseconds) (Snyder and Large, 2005; Fujioka et al., 2009) and macro-scale (e.g., years of genre-specific musical training) (Bianco et al., 2018). For example, it only requires a brief musical sequence to observe beta band activity (14–30 Hz) that increases after each tone, then decreases, creating beta oscillations synchronized to the beat of the music (Fujioka et al., 2009). Gamma band activity (∼30–60 Hz) also increases after each tone, even when a tone that was supposed to occur is omitted, suggesting that gamma oscillations represent an endogenous mechanism of beat anticipation (Fujioka et al., 2009). It was further found that phase-locked, evoked gamma band activity increases about 50 ms after tone onset and diminishes when tones are omitted, showing larger responses during accented beats vs. weak ones, which suggests a neural correlate for meter (Snyder and Large, 2005). Therefore, the aim of our study was to set up a continuous music framework that is not only able to detect encoding of beat and meter, but also able to distinguish between the two.

Tonality is another key component of real-life music listening. We learn what notes or chords will come next in a piece of music based, in part, on the statistical distribution, or frequency, of tones or sequences of tones (Krumhansl and Shepard, 1979). From these observations, Krumhansl and Cuddy (2010) derived the concept of tonal hierarchy, which describes the relative importance of tones in a musical context. By organizing tones in this way, humans assemble a psychological representation of the music based on tonality and rhythm. A few studies have attempted to develop multivariate frameworks that account for this prediction-driven, hierarchical nature of music. For example, Di Liberto et al. (2020) used EEG paired with continuous music stimuli to investigate the relative contributions of acoustic vs. melodic features of music to the cortical encoding of melodic expectation. However, they used monophonic melodies, rather than harmonic, complex music that we would hear in everyday life. They analyzed the EEG data with a useful tool for continuous stimuli, the Multivariate Temporal Response Function (mTRF) MATLAB toolbox, which maps stimulus features to EEG responses by estimating linear transfer functions (Crosse et al., 2016). Sturm et al. (2015) also used ridge regression with temporal embedding to calculate correlations between brain signal and music. Even though they used natural, complex piano music, they chose the power slope of the audio signal as a predictor, which is considered a basic acoustic measure that underlies more complex features such as beat and meter.

Building on the groundwork of these previous multivariate music analyses, we used the mTRF to analyze high-level tonal and rhythmic features of natural, continuous music stimuli extracted with the Music Information Retrieval (MIR) MATLAB toolbox (Lartillot and Toiviainen, 2007). The proposed framework aims to better understand how we process everyday music.

In an attempt to model Krumhansl’s hierarchical organization of musical features, we also expanded on the features provided in the MIR toolbox to further enhance ecological validity. For example, *key clarity*, which measures how tonally similar a given frame of music is to a given key signature, has been used in several studies (Alluri et al., 2012; Sturm et al., 2015; Burunat et al., 2016), yet may not provide an accurate measurement of a musical event’s tonality within the context of the entire musical excerpt. This motivated us to develop a novel feature called *tonal stability*, which contextualizes a particular musical event with respect to the tonal history thus far. *Tonal stability* quantifies the tonal hierarchy of a piece of music by taking the angular similarity between the *key strength* of a certain frame and the averaged *key strength* up until that frame. This allows us to determine how stable a musical event (or a frame) is within a given tonal hierarchy. In other words, it calculates how related the chord implied in an individual frame is to the overarching key, which is derived from a cumulative moving average. By continuously measuring local changes in tonal key centers with respect to the whole musical excerpt, we approximated the ongoing perception of tonal stability. To our knowledge, no prior study has developed such an analytical framework for combining MIR toolbox features with the mTRF to investigate how tonal and rhythmic features are encoded in the EEG signal during the listening of natural music.

We applied our framework to a public domain EEG dataset, the Open Music Imagery Information Retrieval dataset (Stober, 2017), to test the differential cortical encoding of tonal and rhythmic hierarchies. Using model comparisons, we inferred the contribution of individual features in EEG prediction. We show novel ecological evidence confirming and expanding Krumhansl’s theory on how frequency and placement of musical features affect our perception and predictions (Krumhansl and Cuddy, 2010).

## Materials and Methods

The approach we used in the current study is known as linearized modeling of a sensory system (Wu et al., 2006), which has been successfully applied to M/EEG data (Lalor et al., 2006; Di Liberto et al., 2015; Brodbeck et al., 2018) as well as fMRI data (Kay et al., 2008; Huth et al., 2016) in response to naturalistic visual and auditory stimuli. The key idea of the approach is a linearization function (Wu et al., 2006), which captures the nonlinearity of stimulus-response mapping and provides an efficient parameterization of relevant aspects of a stimulus that can be linearly associated with its corresponding response. In this section, we will explain our linearization functions (i.e., musical features), the specifications of the analyzed public data, and practical details of the analysis, which was carried out using MATLAB (RRID:SCR_001622; R2020a) unless otherwise noted.

### Musical Features

We considered a variety of features for the construction of our analytical models. The foundational feature in all models was the temporal *envelope* of the auditory stimulus, which contains low-level acoustic features such as amplitude variations. For tonal hierarchy, we used *key clarity* and *tonal stability* as our two additional features. For rhythmic hierarchy, we looked at the onset of every *beat* and their relative strengths within the given *meter*.

#### Acoustic Feature

A whole-spectrum *envelope* was calculated as the absolute value of the Hilbert transform of the musical signal. The envelope was down-sampled to the EEG’s sampling rate after anti-aliasing high-pass filtering. This feature describing whole-spectrum acoustic energy served as a baseline for other models adding tonal and rhythmic features.

#### Tonal Features

As for high-level tonal features, we computed *key clarity* and *tonal stability*. Key clarity was derived from the MIR toolbox^1^ (v1.7) function mirkeystrength, which computes a 24-dimensional vector of Pearson correlation coefficients corresponding to each of the 24 possible keys (12 major and 12 minor), which is called a *key strength* vector (Gómez, 2006). *Key clarity* is defined by the maximal correlation coefficient, which measures how strongly a certain key is implied in a given frame of interest (Lartillot and Toiviainen, 2007).

Our novel *tonal stability* feature was designed to contextualize the *key strength* with respect to the overall *key strength* of a given musical piece. It is computed with an angular similarity between the *key strength* vector of a single frame and a cumulative average of *key strength* vectors up until the adjacent previous frame as:

(1) $$s\,\left(t\right)=1-\frac{\cos^{-1}\,\cos\,\theta }{\pi }=1-\frac{1}{\pi }\,\cos^{-1}\frac{\text{v}\,\left(t\right)\cdot \bar{\text{v}}\,\left(t-1\right)}{||\text{v}\,\left(t\right)||\cdot ||\bar{\text{v}}\,\left(t-1\right)||}$$

where s(t) is *tonal stability* of the *t*-th frame, is an angle between the two *key strength* vectors, **v**(t) is a *key strength* vector of the *t*-th frame, and $\bar{\text{v}}\,\left(\text{j}\right)=\sum_{i=1}^{j}\text{v}\,\left(i\right)/j$ is a cumulative moving average of *key strength* vectors from the first to the *j*-th frame. The angular similarity is bounded between 0 and 1, inclusively (1 when two vectors are parallel, 0.5 when orthogonal, and 0 when opposite). Thus, the *tonal stability* is also bounded between 0 and 1: 0 when key strength vectors are in opposite directions (i.e., implied keys are most distant on the cycle of fifths; in other words, they share few common tones).

**FIGURE 1:**
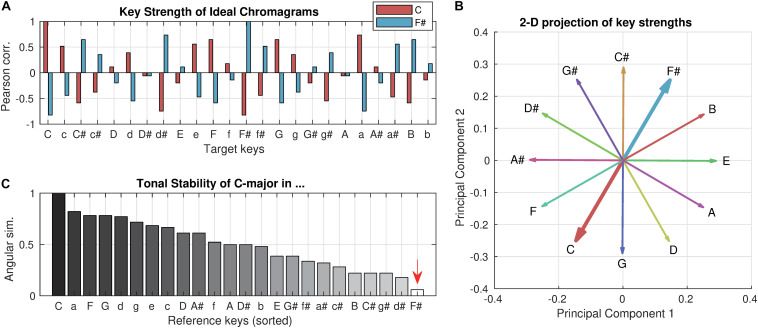
Key strength and tonal stability. **(A)** *Key strength* of the C-major profile (red) and F#-major profile (blue) are computed for all 24 target keys (i.e., Pearson correlation between profiles). The profiles were used as ideal chromagrams that yield a maximal *key clarity* of one. Upper cases represent major keys, and lower cases represent minor keys. **(B)** For geometrical intuition of *tonal stability*, the *key strength* vectors of 12 major keys are projected on the 2-dimensional plane of the first two principal components, which together explained 65% of the total variance. **(C)** *Tonal stability* of the C-major profile with respect to all 24 reference keys (i.e., in all contexts) are computed (i.e., the angular similarity between *key strength* vectors) and sorted in descending order. Note that *key clarity* of the C-major profile is always one while its *tonal stability* varies depending on the reference key (i.e., context).

Using the tonal hierarchy profile (Krumhansl and Shepard, 1979) as an ideal chromagram, which yields a maximal *key strength* of one (Figure 1A), it can be shown that if a chromagram implies a distant key (e.g., C-major key in the F#-major key context), its *tonal stability* would be close to zero. A geometrical appreciation of the relations of *key strength* vectors can be made by a low-dimensional projection using principal component analysis (PCA). The first two principal components explained 65% of the total variance of all *key strength* vectors. When the *key strength* vectors of the 12 major keys are projected to the 2-dimensional plane of the first two principal components (Figure 1B), it becomes clear that the *key strength* vectors of C-major and F#-major are geometrically opposing. Therefore, the (high-dimensional) angular similarity between them would be close to zero (Figure 1C, marked by an arrow; not exactly zero because of higher dimensions that are not visualized), which is our definition of the *tonal stability* feature. On the other hand, the *key clarity* can be seen as the maximal projection to any of the 24 possible dimensions (i.e., maximal intensity projection). Therefore, it is constant regardless of context. In other words, the *tonal stability* quantifies how tonally stable a particular frame is within the context of the entire piece, whereas *key clarity* describes how strongly a tonal structure is implicated in an absolute sense (see Supplementary Figure 1 for an example comparison).

The length of a time window to compute spectrograms should be long enough to cover the lower bound of pitch (i.e., 30 Hz; Pressnitzer et al., 2001) but also not too long to exceed the physiologically relevant spectral range. In the current work, we used a sparse encoding of tonal features based on the estimated beats and measures (see section “Rhythmic Features”). Specifically, at each beat (or measure), a time window was defined from the current to the next beat (or measure). For each time window, the spectrogram, cochleogram, and *key strength* vectors were estimated using the MIR function mirkeystrength, and the *key clarity* and *tonal stability* were calculated as described above. The approach of the sparse encoding is similar to assigning the “semantic dissimilarity” value of a word at the onset in a natural speech study, where N400-like temporal response functions (TRFs) were found (Broderick et al., 2018), and modeling the melodic entropy at the onset of a note (Di Liberto et al., 2020). Previous studies have found an early component (i.e., ERAN; Koelsch et al., 2003) in response to violations within local tonal contexts and a late component (i.e., N400; Zhang et al., 2018) during more global contexts. Therefore, *tonal stability* was expected to be encoded within these latencies.

#### Rhythmic Features

As low-level rhythmic feature, we used *beats* (Grahn and McAuley, 2009; Stober, 2017). *Beats* were extracted using the dynamic beat tracker in the Librosa library^2^ and included in the shared public data. We modeled *beats* using a unit impulse function (i.e., 1’s at beats, 0’s otherwise).

As high-level rhythmic feature, we used *meter*, which was based on *beats*. We weighted the strength of each beat in a musical excerpt, according to a beat accent system that is most prevalent in Western classical music, by separating beats into three tiers: strong, middle, and weak (Grahn and Rowe, 2009; Vuust and Witek, 2014). A separate unit impulse function was created for each of the three levels. Note that the tiers correspond to the strength of a beat, not the position (or phase) within a measure. The breakdown applies as follows:

4/4 meter signature: beat 1=strong; beat 2=weak; beat 3=middle; and beat 4=weak.

3/4 meter signature: beat 1=strong; beat 2=weak; and beat 3=weak.

### OpenMIIR Dataset

We used the public domain Open Music Imagery Information Retrieval Dataset available on Github^3^, which is designed to facilitate music cognition research involving EEG and the extraction of musical features. Given that we only analyzed a subset of the dataset, we will only summarize the relevant materials and methods. Complete details of the experimental procedure can be found in the original study (Stober, 2017).

#### Participants

Data was collected from ten participants. One participant was excluded from the dataset due to coughing and movement-related artifacts, resulting in a total of nine participants. Seven participants were female, and two were male. The average age of the participants was 23. Participants filled out a questionnaire asking about their musical playing and listening background. Seven out of the nine participants were musicians, which was defined as having engaged in a regular, daily practice of a musical instrument (including voice) for one or more years. The average number of years of daily musical practice was 5.4 years. The average number of formal years of musical training was 4.9 years.

Prior to the EEG recording, participants were asked to name and rate how familiar they were with the 12 stimuli of the experiment. Also, before the EEG experiment, they were asked to tap/clap along to the beat, which was then given a score by the researcher based on accuracy. Seven participants were given 100% on their ability to tap along to the beat, and two were given a 92%. All participants were familiar with 80% or more of the musical stimuli.

#### Stimuli

**FIGURE 2:**
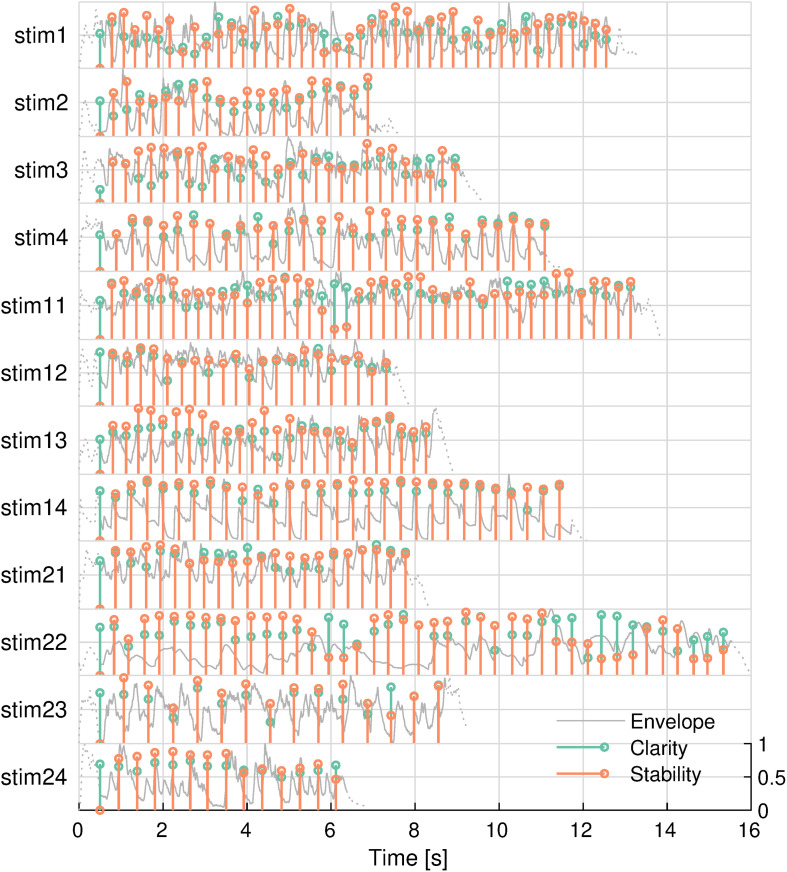
Tonal features of musical stimuli. *Envelope* (gray), *key clarity* (green), and *tonal stability* (orange) are shown. Envelopes outside the analysis window are shown in dashed lines. Stimulus IDs are noted on the left.

There were 12 different, highly familiar musical excerpts that ranged between 6.9 and 13.9 s, with an average duration of 10.5 s each. Exactly half of the songs had a 3/4 time signature, and the other songs had a 4/4 time signature. Table 1 lists the popular songs that the stimuli were taken from. The tonal features of these stimuli are shown in Figure 2. The two features were not significantly correlated in any of the stimuli (minimum uncorrected-*p* = 0.08).

**TABLE 1:** Descriptive statistics of tonal features.

Stim#	Title	Duration (sec)	BPM	BPB	Key clarity (mean ± SD)	Tonal stability (mean ± SD)	Corr. (*p*-value)	Key clarity (mean ± SD)	Tonal stability (mean ± SD)	Corr. (*p*-value)
1	Chim Chim Cheree (lyrics)	13.3	213	3	0.53 ± 0.15	0.63 ± 0.19	0.17 (0.26)	0.52 ± 0.13	0.60 ± 0.24	0.16 (0.57)
2	Take me out to the ballgame (lyrics)	7.7	189	3	0.56 ± 0.14	0.63 ± 0.18	0.19 (0.42)	0.59 ± 0.15	0.60 ± 0.29	0.64 (0.12)
3	Jingle Bells (lyrics)	9.7	200	4	0.51 ± 0.16	0.65 ± 0.18	−0.09 (0.66)	0.55 ± 0.12	0.58 ± 0.28	0.14 (0.73)
4	Mary Had a Little Lamb (lyrics)	11.6	160	4	0.65 ± 0.11	0.68 ± 0.16	0.25 (0.19)	0.67 ± 0.12	0.64 ± 0.28	0.10 (0.81)
11	Chim Chim Cheree (no lyrics)	13.9	206	3	0.69 ± 0.10	0.72 ± 0.21	−0.00 (1.00)	0.70 ± 0.11	0.69 ± 0.27	−0.34 (0.22)
12	Take me out to the ballgame (no lyrics)	7.9	185	3	0.65 ± 0.12	0.69 ± 0.18	0.02 (0.95)	0.71 ± 0.04	0.67 ± 0.28	−0.08 (0.85)
13	Jingle Bells (no lyrics)	9.0	200	4	0.60 ± 0.12	0.72 ± 0.19	0.34 (0.08)	0.54 ± 0.10	0.63 ± 0.31	0.14 (0.76)
14	Mary Had a Little Lamb (no lyrics)	12.2	160	4	0.76 ± 0.09	0.81 ± 0.16	0.15 (0.44)	0.71 ± 0.10	0.78 ± 0.32	−0.33 (0.43)
21	Emperor Waltz	8.3	175	3	0.76 ± 0.11	0.76 ± 0.19	0.14 (0.54)	0.78 ± 0.14	0.71 ± 0.30	−0.15 (0.72)
22	Harry Potter theme	16.0	166	3	0.67 ± 0.16	0.68 ± 0.26	−0.03 (0.84)	0.72 ± 0.13	0.63 ± 0.31	−0.12 (0.69)
23	Star Wars theme	9.2	104	4	0.66 ± 0.16	0.70 ± 0.25	0.19 (0.50)	0.65 ± 0.11	0.68 ± 0.46	0.48 (0.52)
24	Eine Kleine Nachtmusik	6.9	140	4	0.64 ± 0.07	0.67 ± 0.23	0.10 (0.75)	0.69 ± 0.10	0.51 ± 0.36	−0.09 (0.91)

*BPM, beats per minute; BPB, beats per bar; Corr., Pearson correlation coefficient between *key clarity* and *tonal stability*.*

#### Procedure

We analyzed the “Perception” condition, which was the first out of four experimental conditions. The rest of the conditions involved musical imagery tasks, which we did not include in our analysis. Each condition consisted of five blocks. All 12 stimuli were played in a randomized order once per block. This resulted in a total of 60 trials for each condition (i.e., five repetitions per stimulus). In each trial, a stimulus was preceded by two measures of cue beats.

#### Data Acquisition and Preprocessing

Neural signals were measured during the experiment using a BioSemi Active-Two EEG system in 64 channels at a sampling rate of 512 Hz. Independent components associated with ocular and cardiac artifacts were detected using the MNE-python^4^ (RRID:SCR_005972; v0.20.7) (Gramfort et al., 2013), of which demixing matrices were also included in the open dataset. After projecting out the artifact-related components using mne.preprocessing.ica.apply, the EEG signal was converted to handle in EEGLAB^5^ (RRID:SCR_007292; v14.1.2). Then, the data was bandpass-filtered between 1 and 8 Hz using Hamming windowed sinc finite impulse response (FIR) filter using pop_eegfiltnew as the low-frequency activity was previously found to encode music-related information (Di Liberto et al., 2020). Trials were epoched using pop_epoch between 100 ms after music onset (i.e., after beat cues) and 100 ms before music offset with a window length of 200 ms for tonal feature extraction. The EEG signal was then down-sampled to 128 Hz (pop_resample) and normalized by *Z*-scoring each trial.

### mTRF Analysis

#### Model Prediction

The linearized encoding analysis was carried out using the mTRF MATLAB Toolbox^6^ (v2.1) created by Crosse et al. (2016). In a FIR model, we fit a set of lagged stimulus features to response timeseries to estimate time-varying causal impacts of features to the response timeseries:

$$y\,\left(t\right)=\sum_{d=0}^{D}x\,\left(t-d\right)\,b\,\left(d\right)$$

where y(t) and x(t) are a response and a feature at a time point *t*, respectively, and *b*(*d*) is a weight that represents the impact of a feature at a delay *d*. A timeseries of these weights (i.e., a transfer function or a kernel of a linear filter) is called a TRF. In the mTRF encoding analysis, we use a regularized regression (e.g., ridge) to estimate TRFs where multicollinearity exists among multiple features. The encoding analysis is performed at each channel at a time (i.e., multiple independent variables and a univariate dependent variable). The validity of the estimated TRFs is often tested *via* cross-validation (i.e., convolving test features with a kernel estimated from a training set to predict test responses).

When considering multiple features, the FIR model can be expressed in a matrix form:

(2) y=X⁢β+ε

where **y** ∈ *ℝ*^*T* × 1^ is an EEG response vector from a given channel over *T* time points, **X** ∈ *ℝ*^*T* × *PD*^ is a feature matrix of which columns are *P* features lagged over *D* time points (i.e., a Toeplitz matrix), β ∈ *ℝ*^1 × *PD*^ is a vector of unknown weights, and ε ∈ *ℝ*^*T* × 1^ is a vector of Gaussian noise with unknown serial correlation. Note that a feature set could consist of multiple sub-features (e.g., 16-channel cochleogram and 3-channel meter). The vector β is concatenated weights over *D* delays for *P* features. In the current analysis, we column-wise normalized **y** and **X** by taking *Z*-scores per trial.

A ridge solution of Eq. 2 is given (Hoerl and Kennard, 1970) as:

(3) $$\hat{\beta }\,\left(\lambda \right)={\left({\text{X}}^{T}\,\text{X}+\lambda \,\text{I}\right)}^{-1}\,{\text{X}}^{T}\,\text{y},$$

where **I** ∈ *ℝ*^*PD* × *PD*^ is an identity matrix and λ0 is a regularization parameter that penalizes (i.e., shrinks) estimates. That is, the ridge estimates are dependent on the selection of regularization. The lambda was optimized on training data (i.e., a lambda that yields the maximal prediction accuracy for each channel), and the validity of this model was tested on testing data (i.e., predicting EEG response based on given features) through the leave-one-out cross-validation scheme using mTRFcrossval, mTRFtrain, and mTRFevalute. The prediction accuracy was measured by the Pearson correlation coefficient. Specifically, we used 79 delays from −150 ms to 450 ms and 21 loglinearly spaced lambda values from 2^−10^ to 2^10^. We discarded time points where the kernel exceeded trial boundaries (i.e., valid boundary condition) to avoid zero-padding artifacts (e.g., high peaks at zero-lag from short trials).

#### Model Comparison

We created multiple models with varying terms and compared prediction accuracies to infer the significance of encoding of a specific feature in the responses. The families of models were:

(4) $$\text{y}=\left[{\text{X}}_{e\,n\,v}\right]\text{[}\beta_{e\,n\,v}]+\varepsilon$$

(5-1) $$y =\left[{\text{X}}_{env}{\text{ X}}_{beat}\right]\left[\begin{array}{c} \beta_{env}\\ \beta_{beat} \end{array}\right]\,+\,\varepsilon$$

(5-2) $$\text{y = }\left[{\text{X}}_{env}{\text{ X}}_{meter}\right]\left[\begin{array}{c} \beta_{env}\\ \beta_{meter} \end{array}\right]\,+\,\varepsilon$$

(6-1) $$\text{y = }\left[{\text{X}}_{env}{\text{ X}}_{meter}{\text{ X}}_{clarity}\right]\left[\begin{array}{c} \beta_{env}\\ \beta_{meter}\\ \beta_{clarity} \end{array}\right]\,+\,\varepsilon$$

(6-2) $$\text{y = }\left[{\text{X}}_{env}{\text{ X}}_{meter}{\text{ X}}_{stability}\right]\left[\begin{array}{c} \beta_{env}\\ \beta_{meter}\\ \beta_{stability} \end{array}\right]\,+\,\varepsilon$$

where **X_i_** and β**_i_** are a Toeplitz matrix and a weight vector for the *i*-th feature, respectively. Equation 4 served as a baseline model and Eq. 5 are rhythmic models and Eq. 6 are tonal models while covarying rhythmic features. Comparisons of interest were: (a) Eq. 5-1 vs. Eq. 4, (b) Eq. 5-2 vs. Eq. 5-1, (c) Eq. 6-1 vs. Eq. 5-2, and (d) Eq. 6-2 vs. Eq. 5-2. Note that the comparisons were made to infer the effect of the addition of each feature despite their multicollinearity. That is, if there is no uniquely explained variance by the last term, the full model (with the last term) cannot yield greater prediction accuracy than the reduced model (without the last term).

Cluster-based Monte Carlo permutation test (Maris and Oostenveld, 2007), using ft_statistics_montecarlo in FieldTrip^7^ (RRID:SCR_004849; v20180903), was used to calculate cluster-wise *p*-values of one paired *t*-test on differences in prediction accuracies across all channels with the summed *t*-statistics as a cluster statistic. 10,000 permutations with replacement were made to generate null distributions. In permutation tests, a cluster-forming threshold does not affect the family-wise error rate (FWER) but only sensitivity (see Maris, 2019 for formal proof). Thus, clusters were defined at an arbitrary threshold of the alpha-level of 0.05, and the cluster-wise *p*-values are thresholded at the alpha-level of 0.05 to control the FWER to 0.05.

To estimate the variation of the point estimate of a prediction accuracy difference, we bootstrapped cluster-mean prediction accuracies for 10,000 times to compute 95% confidence intervals. For the visualization of results, modified versions of topoplot in EEGLAB and cat_plot_boxplot in CAT12^8^ are used.

#### Control Analysis

To demonstrate the false positive control and the sensitivity of the current procedure, we randomized the phases of envelopes (Menon and Levitin, 2005; Abrams et al., 2013; Farbood et al., 2015; Kaneshiro et al., 2020) to create control features with disrupted temporal structure, but with identical spectra. If the prediction is not due to the encoding of temporal information, this control feature (i.e., phase-randomized envelope) would be expected to explain the EEG data as well as the original envelope. Specifically, the phases of envelopes were randomized *via* fast Fourier transform (FFT) and inverse FFT for each stimulus. That is, within each randomization, the randomized envelope was identical throughout repeated representations over trials. MATLAB’s fft and ifft were used. The phase randomization, model optimization, and model evaluation processes were repeated 50 times across all participants. Then, the prediction accuracies averaged across phase-randomizations were compared with the prediction accuracies with the actual envelopes using the cluster-based Monte Carlo permutation test with the same alpha-levels as in the main analysis.

## Results

### Envelope Tracking

**FIGURE 3:**
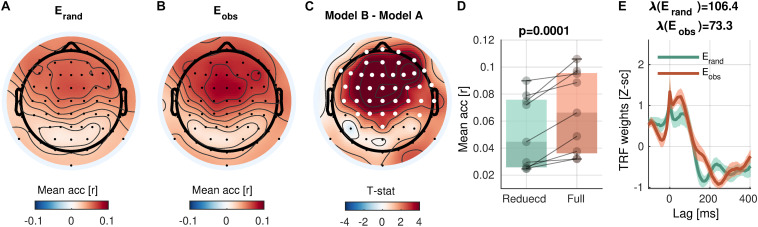
Envelope encoding. **(A,B)** Mean prediction accuracies averaged across subjects are shown in topoplots for a null model (E_rand_, phase-randomized envelope) and a faithful model (E_obs_, observed envelope), respectively. **(C)** *t*-statistics comparing differences in prediction accuracies are shown. Channels included in significant clusters (cluster-*p* < 0.05) are marked in white. **(D)** Prediction accuracies averaged within the cluster with the smallest *p*-value are plotted for each participant. **(E)** Temporal response functions averaged within the cluster are shown. Shades mark one standard error of the mean across participants.

The control analysis revealed that the mTRF analysis sensitively detects envelope tracking compared to models with phase-randomized envelopes (Figure 3). In a cluster with 38 channels over the central and frontal scalp regions, the prediction accuracy with the observed envelopes was significantly higher than randomized envelopes [cluster-mean r_rand_ = 0.0517; r_obs_ = 0.0670; r_obs_ − r_rand_ = 0.0153, 95% CI = (0.0091, 0.0217); summary statistics ΣT = 143.7; cluster-*p* = 0.0001]. As discussed above (see section “Model Comparison”), the higher prediction accuracy of the full model than that of the reduced model (or the null model) indicates that the term of the full model that differs from the reduced (or null) model adds a unique contribution to the prediction, reflecting the neural encoding of the corresponding information. Here, the results suggest that the sound envelope is encoded in the cluster. Note that the peaks at the zero-lag in the TRFs (Figure 3E) are due to the free boundary condition (zero-padding at the boundaries of trials; note that the “condition” here refers to a mathematical constraint and not relevant to experimental conditions), which predicted trial-onset responses in phase-randomized models. When a weaker null model without the trial-onset was compared (i.e., valid boundary condition), the testing revealed increased prediction accuracy in 56 electrodes (cluster-*p* = 0.0001), presumably reflecting the widespread auditory activity *via* volume conduction (figure not shown).

### Rhythmic Hierarchy

**FIGURE 4:**
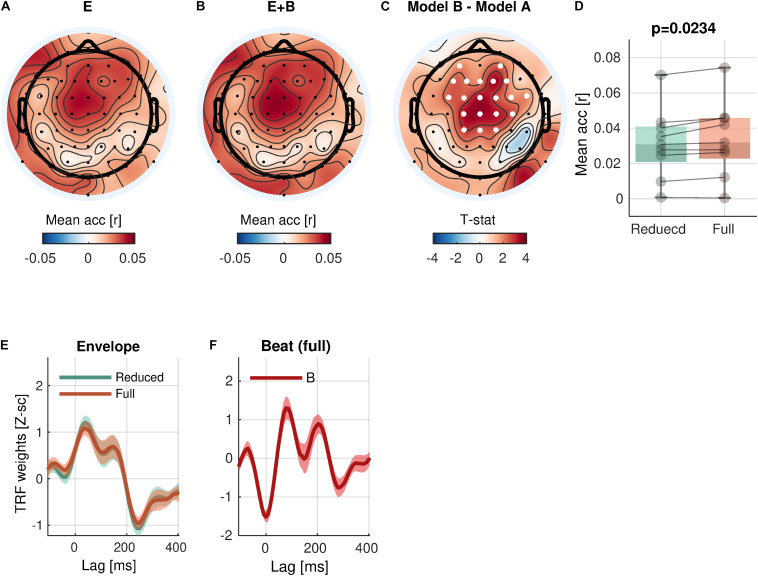
Beat encoding. **(A,B)** Mean prediction accuracies of a reduced model (E, Envelope) and a full model (E + B, Envelope + Beat), respectively. **(C)** *t*-statistics comparing differences in prediction accuracies are shown. Channels included in significant clusters (cluster-*p* < 0.05) are marked in white. **(D)** Prediction accuracies averaged within the cluster with the smallest *p*-value are plotted for each subject. **(E,F)** Temporal response functions of features averaged across electrodes within the cluster are shown. TRFs are *Z*-scored across lags for different regularizations across electrodes/participants.

With respect to the low-level rhythmic feature, the analysis revealed significant encoding of *beat* (Eq. 5-1 vs. Eq. 4; Figure 4) in a cluster of 20 central channels [cluster-mean r_reduced_ = 0.0314; r_full_ = 0.0341; r_full –_ r_reduced_ = 0.0027, 95% CI = (0.0013, 0.0042); ΣT = 52.4; cluster-*p* = 0.0234]. Similarly to the envelope tracking results, a significant increase of prediction accuracy indicates a unique contribution of *beat* in addition to *envelope*.

**FIGURE 5:**
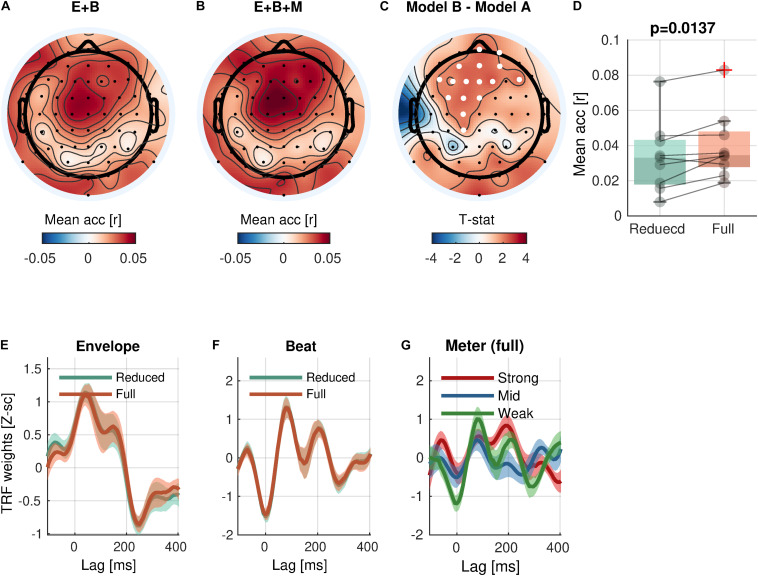
Meter encoding. **(A,B)** Mean prediction accuracies of a reduced model (E + B, Envelope + Beat) and a full model (E + B + M, Envelope + Beat + Meter), respectively. **(C)** *t*-statistics comparing differences in prediction accuracies are shown. Channels included in significant clusters (cluster-*p* < 0.05) are marked in white. **(D)** Prediction accuracies averaged within the cluster with the smallest *p*-value are plotted for each subject. **(E,F)** Temporal response functions of features averaged within the cluster are shown. **(G)** Temporal response functions of the meter features (strong beat, middle beat, and weak beat; e.g., 4/4/: strong-weak-middle-weak) averaged within the cluster are shown. TRFs are *Z*-scored across lags for different regularizations across electrodes/participants.

With respect to the high-level rhythmic feature, the analysis revealed significant encoding of *meter* (Eq. 5-2 vs. Eq. 5-1; Figure 5) in a cluster of 16 frontal and central channels [cluster-mean r_reduced_ = 0.0337; r_full_ = 0.0398; r_full –_ r_reduced_ = 0.0062, (0.0023, 0.0099); ΣT = 34.6; cluster-*p* = 0.0137]. Likewise, a significant increase of prediction accuracy indicates a unique contribution of *meter* in addition to *envelope* and *beat*. The TRFs for *meter* showed different patterns by accents.

### Tonal Hierarchy

We did not find a significant increase of prediction accuracy for either *key clarity* or *tonal stability* calculated on each beat or measure (Eq. 6-1 vs. Eq. 5-2, minimum cluster-*p* = 0.1211; Eq. 6-2 vs. Eq. 5-2, minimum cluster-*p* = 0.0762; Supplementary Figures 2–5).

## Discussion

### Validity of the Proposed Framework

The view that individual elements in natural music may not produce the same effect as they do in isolation is not new. It has been claimed that music is not an objective entity but rather something that is experienced and perceived, raising the need for a dynamic, event-based processing framework (Reybrouck, 2005). The fundamental issue, however, is that often diverse musical features covary to maximize emotional effect (e.g., slow and elegiac melodies in Niccolò Paganini’s Caprice for Solo Violin Op. 1 No. 3 in E minor and energetic arpeggios and triple stops in No. 1 in E major; or subdued, low vocals in Nirvana’s melancholic “Something In The Way” and loud, angry drums in “Smells Like Teen Spirit”). Unless hundreds (if not thousands) of natural stimuli are used (Eerola et al., 2009; Cowen et al., 2020), it is impossible to tease out the effect of one element (or an independent component of elements) from another with a small number of stimuli. For this reason, it has been an established tradition to isolate and orthogonalize musical features or acoustic properties to study their effects in music psychology and cognitive neuroscience of music. However, now that computational models can translate naturalistic stimuli into relevant features (i.e., linearizing functions), recent human neuroimaging studies have shown that it is possible to analyze complex interactions among natural features while taking advantage of the salience of naturalistic stimuli to evoke intense emotions and provide ecologically valid contexts (Goldberg et al., 2014; Sonkusare et al., 2019; Jääskeläinen et al., 2021).

In the current study, we demonstrated a simple yet powerful framework of a linearized encoding analysis by combining the MIR toolbox (a battery of model-based features) and mTRF Toolbox (FIR modeling with ridge regression). First, we showed that ridge regression successfully predicted envelope-triggered cortical responses in the ongoing EEG signal in comparison to null models with a phase-randomized envelope. Furthermore, our proposed framework detected cortical encoding of rhythmic, but not tonal, features while listening to naturalistic music. In addition, the estimated transfer functions and the spatial distribution of the prediction accuracies made neuroscientific interpretations readily available. These findings differentiate themselves from previous studies using similar regression analyses that only used either monophonic music or simple, low-level acoustic features, such as note onset (Sturm et al., 2015; Di Liberto et al., 2020).

### Cortical Encoding of Musical Features

We showed cortical encoding of beats and meter during the listening of every day, continuous musical examples. This was observed most strongly over frontal and central EEG channels, which have long been implicated as markers of auditory processing activity (Näätänen and Picton, 1987; Zouridakis et al., 1998; Stropahl et al., 2018). However, *key clarity* and *tonal stability* were not conclusively represented in the cortical signal in our models.

Unlike the tonal features, both of the rhythmic features (*beat* and *meter*) were encoded strongly in the neural signal. The TRF for *beat* showed a steady periodic signal, consistent with the finding in the original OpenMIIR dataset publication by Stober (2017) that the peaks of the event-related potentials (ERPs) corresponded to the beat of the music. This means that both the ERPs in the study by Stober (2017) and our *beat* TRFs displayed large peaks at zero-lag, implying that beats may be anticipated. The possibility of an anticipatory mechanism of beats is consistent with the view that humans may possess an endogenous mechanism of beat anticipation that is active even when tones are unexpectedly omitted (Fujioka et al., 2009). The relatively early latency of the additional TRF peaks between 100 and 200 ms suggests that beats may be processed in a bottom-up fashion as well. Humans engage in an active search for the beat when it becomes less predictable by adaptively shifting their predictions based on the saliency of the beats in the music, suggesting that beats also provide useful exogenous cues (Toiviainen et al., 2020). The use of continuous music and EEG in the proposed framework lends itself particularly well to determining these various mechanisms of beat perception.

It has also been shown that different populations of neurons entrain to beats and meter (Nozaradan et al., 2017). Moreover, phase-locked gamma band activity has further suggested a unique neural correlate to meter (Snyder and Large, 2005). Extending these previous findings, the current results in the low frequency band (1–8 Hz) revealed this dichotomy between beats and meter through their different topologies. *Beat* was encoded over a tight cluster of central channels, but *meter* was encoded over a large cluster of frontal channels. The significant increase in prediction accuracy observed over widespread frontal channels for *meter* might suggest a distant source although it is not possible to uniquely determine the source location only from the sensor topography (i.e., inverse problem). That is, the topography also could be due to widely spread but synchronized cortical sources. However, there is evidence based on deep brain stimulation and scalp recording that EEG is sensitive to subcortical sources (Seeber et al., 2019). The putamen, in particular, has been proposed as a region of meter entrainment, while the cortical supplementary motor area is more associated with beats (Nozaradan et al., 2017; Li et al., 2019). The distinct topologies observed between the beats and meter features are especially intriguing given the relatively short duration of each stimulus (10.5 s on average).

It was unexpected that neither of the tonal features was significantly correlated with the EEG signal, given that previous studies suggested that information about these tonal structures is reflected in non-invasive neural recordings. For instance, previous ERP studies showed stronger responses to deviant harmonies than normative ones (Besson and Faïta, 1995; Janata, 1995; Koelsch et al., 2003). Additionally, in a recent MEG study (Sankaran et al., 2020), a representational similarity analysis revealed that distinctive cortical activity patterns at the early stage (around 200 ms) reflected the absolute pitch (i.e., fundamental frequencies) of presented tones, whereas late stages (after 200 ms onward) reflected their relative pitch with respect to the established tonal context (i.e., tonal hierarchy) during the listening of isolated chord sequences and probe tones played by a synthesized piano. In a study with more naturalistic musical stimuli (Di Liberto et al., 2020), the cortical encoding of melodic expectation, which is defined by how surprising a pitch or note onset is within a given melody, was shown using EEG and the TRF during the listening of monophonic MIDI piano excerpts generated from J. S. Bach Chorales. With respect to *key clarity*, it was shown that *key clarity* correlates significantly with behavioral ratings (Eerola, 2012) and is anti-correlated with the fMRI signal timeseries in specific brain regions, including the Rolandic Operculum, insula, and precentral gyrus, while listening to modern Argentine Tango (Alluri et al., 2012). In a replication study with identical stimuli (Burunat et al., 2016), *key clarity* showed scattered encoding patterns across all brain regions with weaker magnitudes of correlations, although such an association with evoked EEG responses (or the absence thereof) has not been previously reported. One possibility for the current negative finding with respect to tonal features is that the musical stimuli in the current dataset might not have been optimal for our interest in the tonal analysis given their tonal simplicity (see section “Limitations” for further discussion).

### Limitations

The stimuli were relatively short in duration (10-s long on average) and often repetitious in nature. These stimulus characteristics limited the ability to observe the response to larger changes in *key clarity* and *tonal stability*. For instance, the ranges of standard deviation of *key clarity* and *tonal stability* were (0.0667, 0.1638) and (0.1570, 0.2607), respectively, when calculated on beats. These were narrower than typical musical stimulus sets [e.g., 360 emotional soundtrack 15-s excerpts (Eerola and Vuoskoski, 2011); (0.0423, 0.2303) and (0.11882, 0.3441) for *key clarity* and *tonal stability*, respectively]. These limitations (short lengths and limited variation in tonality) might have contributed to negative findings in the current study. Another limitation in the dataset was the small number of participants (*n* = 9), which limited statistical power. Future neuro-music public datasets (e.g., the one developed by Grahn et al., 2018) may want to consider using longer, more dynamic musical excerpts, especially ones that have increased dramatic shifts in tonality with more participants. The dataset also did not contain simultaneous behavioral ratings of the music, which resulted in us being unable to analyze our data alongside measures such as emotion.

One limitation in our analysis is that we used a single regularization parameter for all features, as currently implemented in the mTRF Toolbox. However, it has been shown that using independent regularization for each feature set (“banded ridge”) can improve the prediction and interpretability of joint modeling in fMRI encoding analysis (Nunez-Elizalde et al., 2019). Thus, it is expected that a systematic investigation on the merits of banded ridge regression in mTRF analysis on M/EEG data would benefit the community.

## Future Directions and Conclusion

Ultimately, we hope that this framework can serve two broad purposes. The first is for it to enhance the ecological validity of future music experiments. The second is for it to be used as a tool that can be paired with other metrics of interest. Emotion is perhaps the most fitting application of this framework, given the special ability of music to make us experience intense feelings. Combining the current analytic framework with behavioral measures like emotion will be especially useful because it could shed light on what factors interact with our anticipation of tonality and rhythm during music listening. In particular, when combined with continuous behavioral measures, such as emotion or tension, this might 1 day be used to elucidate how changes in certain musical features make us happy or sad, which could deepen our knowledge of how music can be used therapeutically or clinically. Furthermore, some current limitations of the *tonal stability* measure provide future researchers with opportunities for innovation. Looking forward, it would be useful to create a *tonal stability* measure that can account for multiple (shifting) tonal centers within a single piece of music.

In summary, we presented an analytical framework to investigate tonal and rhythmic hierarchy encoded in neural signals while listening to homophonic music. Though the model did not demonstrate the presence of the proposed *tonal stability* measure, it did successfully capture cortical encoding of rhythmic hierarchy. Moreover, the framework was able to differentiate the spatial encoding of low/high-level features, as represented by the separate encoding of beat and meter, suggesting distinct neural processes. The current framework is applicable to any form of music by directly feeding audio signals into the linearizing model. In addition, it has the possibility of including other time-resolved measures to appropriately address the complexity and multivariate nature of music and other affective naturalistic stimuli. This will bring us to a more complete understanding of how tonality and rhythm are processed over time and why the anticipation and perception of these features can induce a variety of emotional responses within us.

## Data Availability Statement

The datasets presented in this study can be found in online repositories. The names of the repository/repositories and accession number(s) can be found in the article/Supplementary Material.

## Ethics Statement

The studies involving human participants were reviewed and approved by the Ethics Board at the University of Western Ontario. The patients/participants provided their written informed consent to participate in this study.

## Author Contributions

JL and S-GK conceived the ideas, developed the analytic framework, analyzed the public data, and wrote the first draft together. S-GK formulated models and wrote code for analysis and visualization. JW and TO contributed to conceiving ideas, interpreting results, and writing the manuscript. All authors contributed to the article and approved the submitted version.

## Conflict of Interest

The authors declare that the research was conducted in the absence of any commercial or financial relationships that could be construed as a potential conflict of interest.

## Footnotes

1 http://bit.ly/mirtoolbox

2 https://github.com/bmcfee/librosa

3 https://github.com/sstober/openmiir

4 https://mne.tools/stable/index.html

5 https://sccn.ucsd.edu/eeglab/index.php

6 https://github.com/mickcrosse/mTRF-Toolbox

7 https://www.fieldtriptoolbox.org/

8 http://www.neuro.uni-jena.de/cat/
