# Supplementary material for: An Analytical Framework of Tonal and Rhythmic Hierarchy in Natural Music Using the Multivariate Temporal Response Function
Source: Front Neurosci. 2021 Jul 16;15:665767. doi: 10.3389/fnins.2021.665767 (PMC8322238; doi:10.3389/fnins.2021.665767)
Supplement: Supplementary file 1 [file Data_Sheet_1.PDF]

Supplementary Material

1 Supplementary Figures

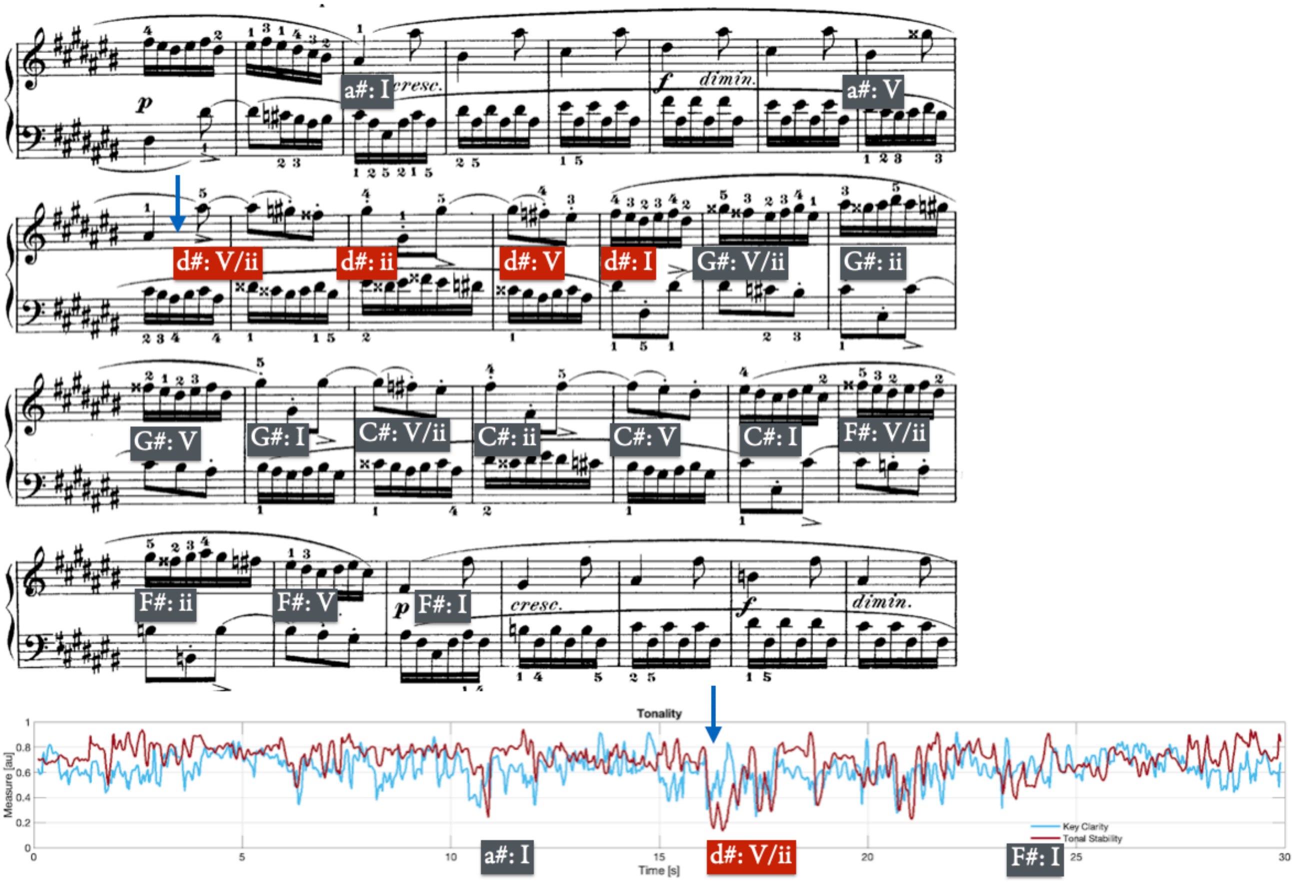

Supplementary Figure S1. **Key clarity and tonal stability**. As an example comparison, key clarity (blue) and tonal stability (red) were calculated for a 30-s excerpt from J. S. Bach's Prelude and Fugue in C# major, BWV 848 with 50% overlapping 200-ms frames. A modulation to a key (D# minor) that is distant from the overall key of the excerpt (C# major) was detected by a sudden decrease in tonal stability (marked with blue arrows), whereas key clarity was insensitive to such tonal relationships. The musical score is in the public domain[1].

[1] https://imslp.org/wiki/Prelude_and_Fugue_in_C-sharp_major,_BWV_848_(Bach,_Johann_Sebastian)

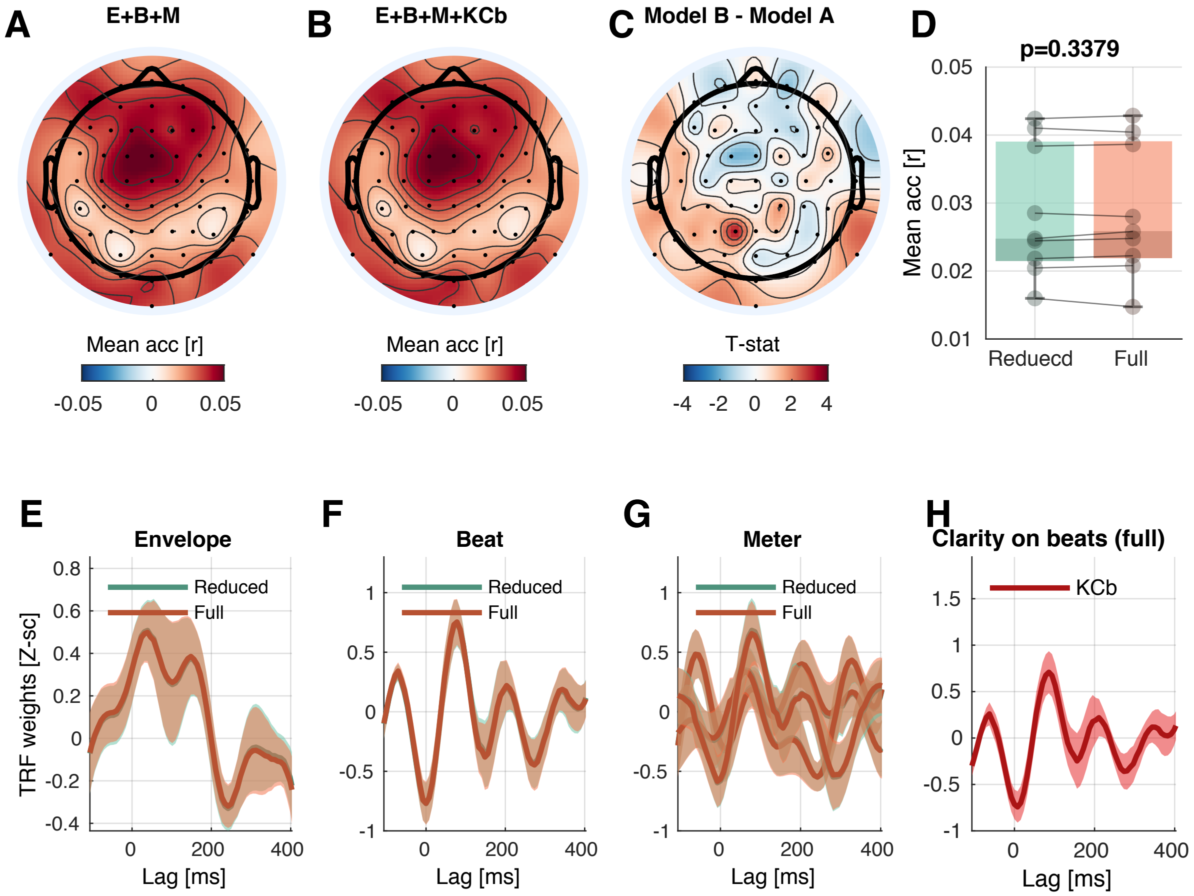

Supplementary Figure S2. **Key-clarity-on-beat encoding**. (A, B) Mean prediction accuracies of a reduced model (E+B+M: Envelope + Beat + Meter) and a full model (E+B+M+KCb: Envelope + Beat + Meter + Key clarity on beats), respectively. (C) T-statistics comparing differences in prediction accuracies are shown. (D) Prediction accuracies averaged across all channels are plotted for each subject. (E–H) Temporal response functions of features averaged across all channels are shown. TRFs are Z-scored across lags for different regularizations across electrodes/subjects.

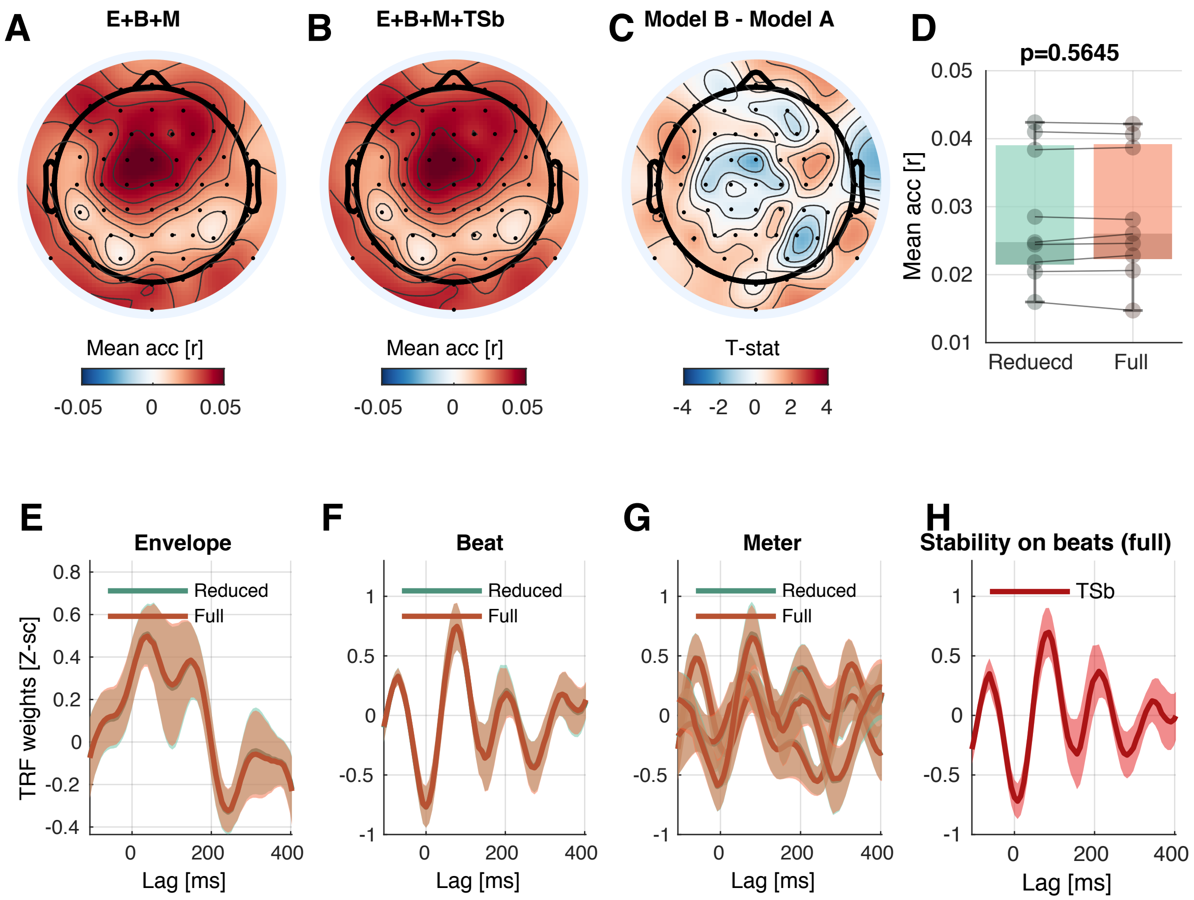

Supplementary Figure S3. **Tonal-stability-on-beat encoding**. (A, B) Mean prediction accuracies of a reduced model (E+B+M: Envelope + Beat + Meter) and a full model (E+B+M+TSb: Envelope + Beat + Meter + Tonal stability on beats), respectively. (C) T-statistics comparing differences in prediction accuracies are shown. (D) Prediction accuracies averaged across all channels are plotted for each subject. (E–H) Temporal response functions of features averaged across all channels are shown. TRFs are Z-scored across lags for different regularizations across electrodes/subjects.

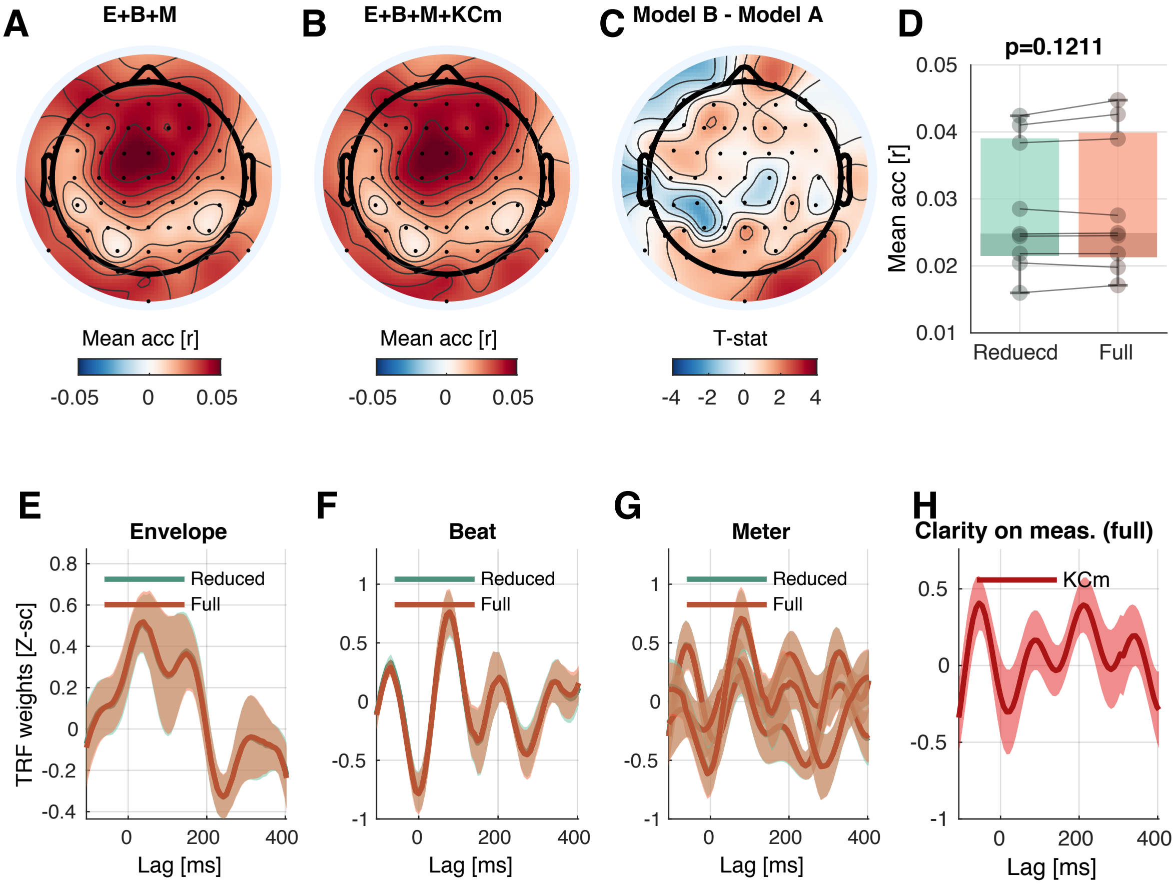

Supplementary Figure S4. **Key-clarity-on-measure encoding**. (A, B) Mean prediction accuracies of a reduced model (E+B+M: Envelope + Beat + Meter) and a full model (E+B+M+KCm: Envelope + Beat + Meter + Key clarity on measures), respectively. (C) T-statistics comparing differences in prediction accuracies are shown. (D) Prediction accuracies averaged across all channels are plotted for each subject. (E–H) Temporal response functions of features averaged across all channels are shown. TRFs are Z-scored across lags for different regularizations across electrodes/subjects.

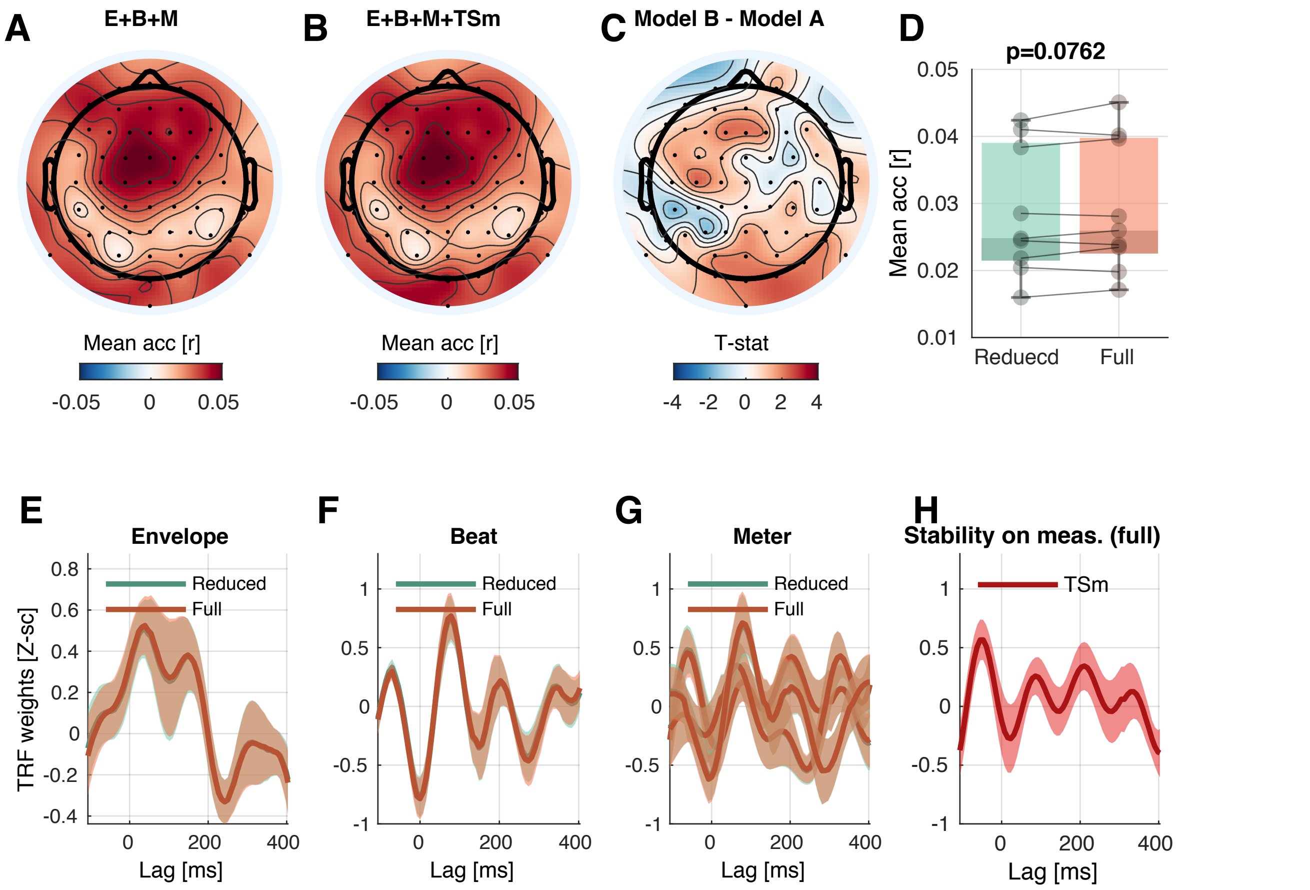

Supplementary Figure S5. **Tonal-stability-on-measure encoding**. (A, B) Mean prediction accuracies of a reduced model (E+B+M: Envelope + Beat + Meter) and a full model (E+B+M+TSm: Envelope + Beat + Meter + Tonal stability on measures), respectively. (C) T-statistics comparing differences in prediction accuracies are shown. (D) Prediction accuracies averaged across all channels are plotted for each subject. (E–H) Temporal response functions of features averaged across all channels are shown. TRFs are Z-scored across lags for different regularizations across electrodes/subjects.
